# Gametogenesis From the Early History Life Stages of the Kumamoto Oyster *Crassostrea sikamea* and Their Breeding Potential Evaluation

**DOI:** 10.3389/fphys.2019.00524

**Published:** 2019-05-15

**Authors:** Yuehuan Zhang, Yanping Qin, Lai Ma, Zihua Zhou, Shu Xiao, Haitao Ma, Ying Pan, Jun Li, Ziniu Yu

**Affiliations:** ^1^Key Laboratory of Tropical Marine Bio-resources and Ecology, South China Sea Institute of Oceanology, Chinese Academy of Sciences, Guangzhou, China; ^2^Guangdong Provincial Key Laboratory of Applied Marine Biology, Guangzhou, China; ^3^Beihai Southern Ocean Ecological Culture Limited, Beihai, China; ^4^College of Animal Science and Technology, Guangxi University, Nanning, China

**Keywords:** Kumamoto oyster, *Crassostrea sikamea*, sex differentiation, physiological maturity, functional maturity, breeding potential

## Abstract

The Kumamoto oyster, *Crassostrea sikamea*, is native to Southeast Asia, including China, Japan and Korea, and is an important traditional wild fishery resource. Although this oyster’s early gametogenesis was reported in Mexico, no related research was found on the breeding potential using early forming gametes. We re-examined the gametogenesis of *C. sikamea* during early life history in southern China and further divided it into three phases: sex differentiation (1 month old, shell height 2–3 mm), physiological maturity (2 months old, shell height 3–5 mm) and functional maturity (3 months old, shell height 9–12 mm). The breeding potential was evaluated using four sets of gametes from parent oysters of different ages (2, 3, 6, and 15 months old). The physiologically mature gametes were not suitable for artificial hatchery due to the low production of eggs, and yielding a high deformity rate of D larvae (95.47 ± 1.25%) and heavy larval morality (90.23 ± 1.84%) post-fertilization. However, progeny from functionally mature gametes grew significantly faster than those of other age groups, with no significant differences in fertilization, hatching level or survival of progeny among them. This study clearly demonstrates that the first batch of functionally mature gametes can develop normally and produce viable progeny, suggesting that artificial hatchery of *C. sikamea* is completely feasible using parent oysters from 3 months old and onward. Furthermore, this hatchery method can effectively shorten the breeding cycle and accelerate the breeding process.

## Introduction

The reproductive strategies of an organism play a major role in the dynamics of the population and the biogeography and continuity of the species. Prior to the offspring production, reproduction process involve numerous biological development process with spermatogenesis and oogenesis as fundamental phase of gametogenesis (Camacho-Mondragón et al., 2015). The process also involves in differences reserve energy expenditure pattern for growth and reproduction that allow animal to continue grow and reproduce healthily at different life stages as a survival strategy that have been developed in marine invertebrates (Llodra, 2002).

The Kumamoto oyster *Crassostrea sikamea* is widely distributed throughout Southeast Asia, including in Japan (Ariake Sea and Seto Inland Sea) (Hedgecock et al., 1993; Hamaguchi et al., 2013), Korea (Suncheon Bay) (Hong et al., 2012) and China (Southern China) (Wang et al., 2013). It occurs not only naturally in China, but also lives in abundance over a wide geographical distribution, ranging from Jiangsu to Guangxi, including Hainan Island (Wang et al., 2013). Traditionally, this oyster was a wild fishery resource; farmers capture these oysters from the reef or stones in the intertidal zone. Recently, the wild resource has sharply declined due to environmental pollution and marine land reclamation, so the artificial hatching and culture of *C. sikamea* was in demand to increase oyster yield (Zhang et al., 2016).

The key biological factors influencing the Kumamoto oyster reproduction are broodstock sizes and ages (Cáceres-Martínez et al., 2012). The understanding of the parents’ biology is critical in determining the availability of broodstock for spawning, the seedling production success and the length of breeding cycles. In the present study, we re-examined the stages of gametogenesis during the early life phases of *C. sikamea*, and evaluated the breeding potential of these early forming gametes by comparing the phenotypic traits of progeny from parents aged between 2 and 15 months old. We further divided it into three phases: sex differentiation (1 month old, shell height 2–3 mm), physiological maturity (2 months old, shell height 3–5 mm) and functional maturity (3 months old, shell height 9–12 mm). This study can provide a new scientific selective standard of Kumamoto oyster broodstock for seed production, and can be applied to genetic breeding field.

## Materials and Methods

### Gametogenesis

**FIGURE 1:**
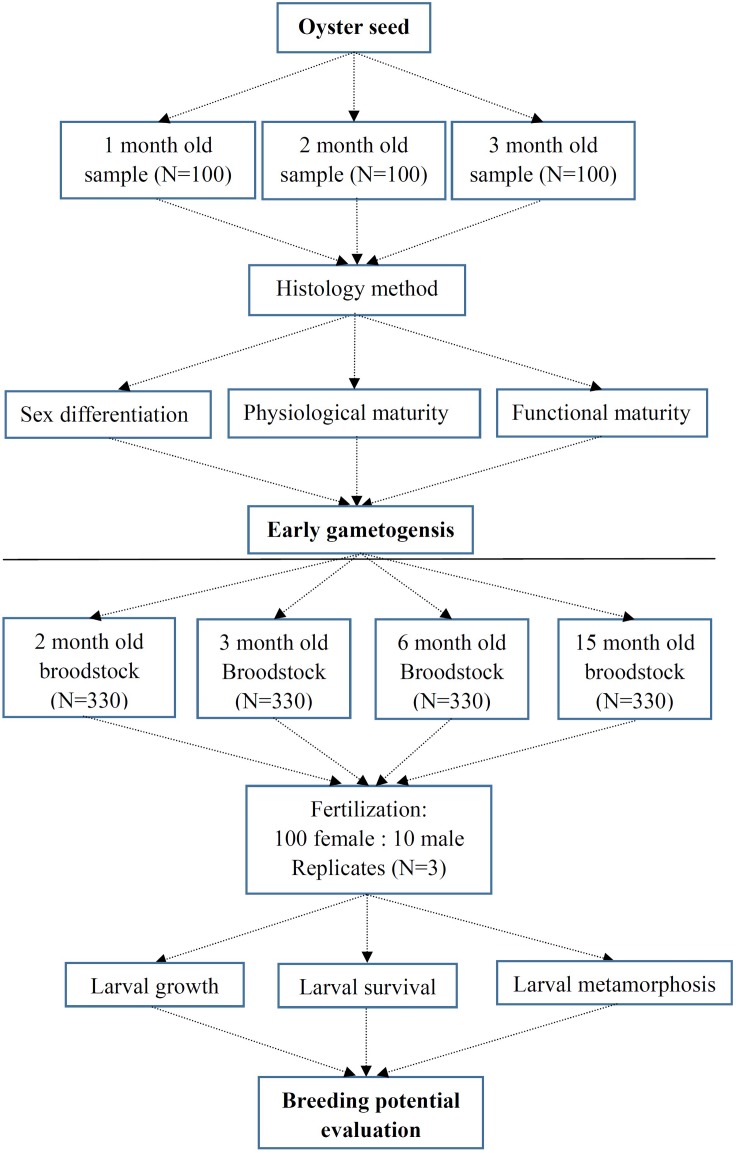
Experimental protocol of early gametogensis of *Crassostrea sikamea* and their breeding potential evaluation.

The mature broodstock of Kumamoto oyster *C. sikamea* were originally collected from Zhanjiang (Guangdong, China) at Qintang Town (110.621894E, 21.226126N) and their status was confirmed by the molecular markers (Wang and Guo, 2008) in May 2013. Artificially hatched progeny of *C. sikamea* were examined during the first three months of their early life, where three sets of samples were collected at 30, 60, and 90 days, respectively, after fertilization. Oyster samples were cleaned using running water to remove debris. The shell height of all oysters were measured using an electronic caliper (LINKS, 0.01 mm). All oysters samples were placed in Bouin’s fluid for 24 h and then preserved in 70% alcohol for histology section (Enríquez-Díaz et al., 2009). Subsequently, specimens were dehydrated with ethanol, and embedded in paraffin. Standard 6-μm sections were stained with Hematoxylin and Eosin for optical observation. Gametogenesis levels during the early stages were determined using the protocol for *C. gigas* (Normand et al., 2008) and *C. sikamea* (Cáceres-Martínez et al., 2012). Early gametogensis protocol of *C. sikamea* was described by Figure 1.

### Artificial Hatchery

Gametes from four sets of broodstocks with varying parental ages (2-, 3-, 6- and 15-month old parents, Table 1) by artificial hatching were obtained by dissection in October 2014. Each experimental age group consisted of three replicates. For each group, eggs from 100 females were pooled, and then fertilized with pooled sperm from ten males within 60 min at a density of approximately 5–8 sperm per egg. In total, approximately 90 × 10^4^ eggs from 1.2 liters of seawater were used for each experimental group and the remaining eggs from each replicate were abandoned. Fertilized eggs were sampled and kept in beakers to evaluate the fertilization. Fertilization was conducted at 27.8–28.2°C in sand filtered seawater with a salinity of 20 ppt.

**TABLE 1:** Age, size and fecundity of different old oyster broodstocks.

Age (months)	Shell height (mm)	Fresh weight g/ind.	Fecundity × 10^4^ eggs/ind.
2	3–5	0.1–0.2	<0.1
3	9–12	1.0–1.5	3–5
6	24–30	4.5–7.0	120–180
15	40–60	15.0–27.5	600–900

After 20 h fertilization, D-larvae from each replicate were collected on a 40-μm screen and reared in three 100 L buckets at concentrations of 2–3 larvae/mL with aeration. Larvae were fed with *Isochrysis zhanjiangensis* on days 0–6, and a mixture of wholly diatom species *Nannochloropsis oculata* after day 6. Feeding was increased gradually from 6,000 to 80,000 cells/mL/day during 3 weeks (Zhang et al., 2016). Seawater was exchanged 50% every day. The following water quality parameters were maintained: temperature at 27.6–28.9°C, salinity at 20 ppt, and pH at 7.83–8.20.

When over 60% larvae developed an eye spot and foot, they were placed in pools with settlement substrate by cleaning at a density of 2 shells/L. Larvae set within 8 days, and newly settled spat were nursed for 9 weeks and fed 80,000–100,000 cells/mL/day of a 1:1 mixture of *Chlorella vulgaris* and *Nannochloropsis oculata* (volume ratio). Water was exchanged new filtered seawater 30% once daily. Breeding potential of gametes from *C. sikamea* was designed by Figure 1.

### Index Measurements

The embryo development (egg diameter, fertilization rate and hatching rate), D larvae ratio was the percent value by calculating the number fertilized eggs to D larvae; Larval shell height was measured by the optical microscope (Olympus, CX33); survival rate, growth rate and metamorphic index of each group were evaluated. Larvae survival rate was taken as the ratio of the number of eye larvae to the number of D larvae; larvae growth rate was the daily increase in larval shell height; the metamorphic time was defined as the duration length of time between the appearance of D larvae to 90% larval settlement; the metamorphic rate was the ratio of the number of spat to the number of eye larvae; and the metamorphic size was the maximum primary shell height.

### Data Analysis

Differences in phenotypic traits between groups and replicates were analyzed using multiple comparisons of means using one-way ANOVA. To improve the normality and homoscedasticity, the fertilization rate, hatching rate, survival rate and metamorphic rate were arcsine transformed prior to analysis, and the size of egg diameter, D larvae and metamorphic size and metamorphic time were logarithmically transformed (base 10) (Parker et al., 2009). All statistical analyses were performed using SPSS19.0 and significance for all analyses was set to *P* < 0.05 unless noted otherwise.

## Results

### Early Gametogenesis

The gonads of the oyster are scattered throughout the visceral mass and their absolute size varies depending on the number and diameter of gametes enclosed. The gametogenesis process throughout the first 3 months of life was divided into three phases: sex differentiation, physiological maturity and functional maturity.

### Sex Differentiation

**FIGURE 2:**
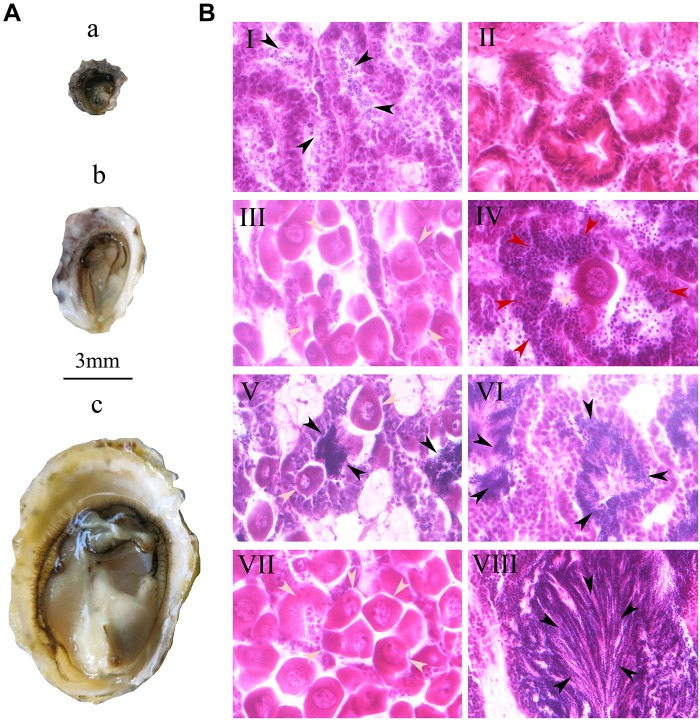
Gonad of oyster *Crassostrea sikamea* **(A)** and gametogenesis **(B)** during the early life history at the first three months stage. In plane **A**: a, One month old oyster; b, Two month old oyster; c: Three month old oyster. In plane B: I, Male at one month; II, Asexuality at one month; III, Female at two month; IV, Hermaphrodite (spermatocyte and oocyte) at two month; V Hermaphrodite (Sperm and oocyte) at two month; VI, Male at two month; VII, Female at three month; VIII, Male at three month. In panels **B**, the gray, black, and red arrows indicate oocytes, sperms and spermatocytes, respectively. All histological section pictures were taken under multiple of ×40.

At 1 month, the shell height of oysters ranged from 1.5 to 3.0 mm, with a mean value of 2.48 mm (±0.36) and no obvious gonadal development in the visceral mass (Figure 2Aa). 27.0% of individuals developed reproductive follicles with sperm (Figure 2BI), while in the other 73% there was no sexual differentiation visible by histological section (Figure 2BII and Table 2). During the process of sexual differentiation, the primary gonad of *C. sikamea* contains the antecedent cells of both sexes when very young, and its protandric trait were recognized as the rapid proliferation of spermatogonia and then formation of sperm.

**TABLE 2:** Sex ratio of 1.0, 2.0, and 3.0 month old *C. sikamea* during the first three months of life history.

Item	Replicate	Female	Male	Hermaphroditism	Asexuality	Total
1.0 month	1	–	25	–	75	100
	2	–	24	–	76	100
	3	–	32	–	68	100
	**Mean**	**–**	**27.0**	**–**	**73.0**	**–**
2.0 month	1	12	79	1	8	100
	2	10	83	0	7	100
	3	8	82	1	9	100
	**Mean**	**10.0**	**81.3**	**0.7**	**8.0**	**–**
3.0 month	1	35	65	–	–	100
	2	24	76	–	–	100
	3	26	74	–	–	100
	**Mean**	**28.3**	**71.7**	**–**	**–**	**–**

### Physiological Maturity

At 2 months, the shell height of the 90 oysters ranged from 3 to 5 mm, with a mean value of 3.96 mm (±0.57) with a small amount of gonadal development in the visceral mass (Figure 2Ab). 10.0, 0.7, 81.3, and 8.0% of the oysters were female, hermaphroditic, male and asexual, respectively, suggesting that the physiological maturity stage had begun (Figures 2BIII,VI and Table 2). At this stage, oocyte diameters in females ranged from 27 to 40 μm, and they were not of uniform size (Figure 2BIII). There were two types of hermaphrodite, one type with oocytes and spermatocytes and another type with oocytes and sperm (Figure 2BV). A certain number of sperm were found in the male gonads (Figure 2BVI). Sex differentiation still had not occurred in asexual individuals. Physiological maturity of bivalves is defined as the point at which the gametes in the gonad attain their mature size and morphology. However, this stage does not mean that the individual actually participates in spawning behavior. Although we were able to observe gametes by histology section, very few eggs were obtained from soft body by dissection for hatchery.

### Functional Maturity

At 3 months, the shell height of the 90 oysters ranged from 9 to 12 mm, with a mean value of 10.78 mm (±1.25). All oysters had fully mature gonads and gametes (Figure 2Ac), and were either female or male; no hermaphroditism or asexuality was observed. 28.3% of oysters were female and 71.1% were male (Figures 2BVII,VIII and Table 2), meaning that they had reached the functionally mature stage. At this stage, the females’ oocytes were close to 40 μm in size with consistent diameters, while in the males large quantities of sperm were present in the seminal vesicles. Functional (physical) maturity is defined as the point at which the oysters enter the first spawning stage, when the males and females spontaneously release sperms and eggs, respectively.

### Hatchery

**FIGURE 3:**
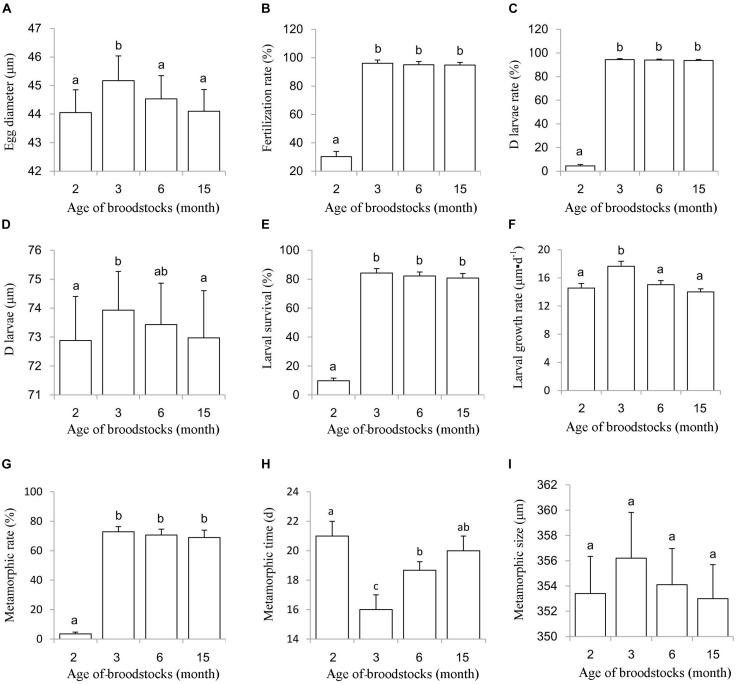
Hatching index, larval survival, growth, metamorphosis of progeny from 2, 3, 6, and 15 month old parents. **(A)** Egg diameter; **(B)** Fertilization rate; **(C,D)** larvae rate; **(D)** D larvae size; **(E)** Larval survival rate; **(F)** Larval growth rate; **(G)** Metamorphic rate; **(H)** Metamorphic time; **(I)** Metamorphic size.

**FIGURE 4:**
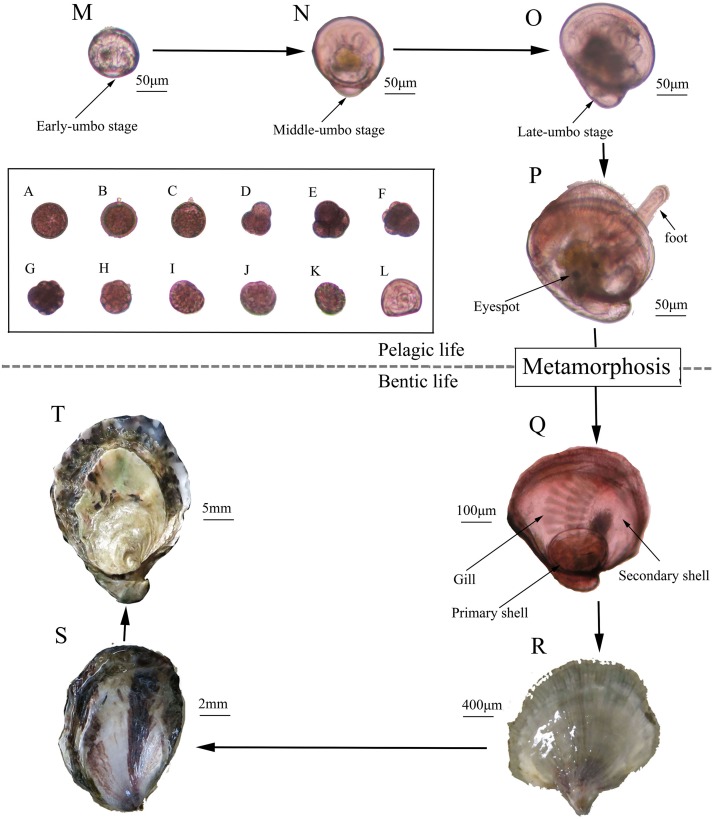
History life cycle of the Kumamoto oyster. **(A)** Fertilized egg; **(B)** First polar; **(C)** Second polar; **(D)** Two cells; **(E)** Four cells; **(F)** Eight cells; **(G)** Sixteen cells; **(H)** Multi-cells; **(I)** Mulberry; **(J)** Blastocyst; **(K)** Trochophore; **(L)** D larvae; **(M)** Early umbo-larvae; **(N)** Middle umbo-larvae; **(O)** Late umbo-larvae; **(P)** Eye larvae; **(Q)** New forming spat; **(R)** Spat; **(S)** Youth; **(T)** Adult.

To assess whether these early gametes are fully functional, we conducted an artificial hatchery experiment using 2- and 3-month-old oyster parents, with 6- and 15-month old oyster parents as control groups. The average diameter of the eggs from the 3-month group (45.17 μm) was significantly larger than that of those from the other three groups (*P* < 0.05) (Figure 3A). The fertilization rate (30.27%) and D larval rate (4.53%) for the 2-month group were significant lower than the other three groups (*P* < 0.05), while fertilization rate and D larvae for the 3-, 6- and 15-month groups all exceeded 90%, with no significant differences among these three groups (*P* > 0.05) (Figures 3B,C). Normal embryo development, including fertilization, first polar stage, second polar stage, 2 cell stage, 4 cell stage, 8 cell stage, 16 cell stage, and multi-cell stage, mulberry stage, blastocyst stage, trochophore stage and D larvae (Figures 4A–L), was observed, and the duration of development from fertilization to D larvae was approximately 20 h for all four groups, which were kept at a temperature of 27.8–28.2°C with a salinity of 20 ppt. D larvae of shell length from the 2-, 3-, 6-, and 15-month groups were 72.88, 73.93, 73.43, and 72.97 μm, respectively. No significant difference was found between the D larval size of 6-month group and the other three groups (*P* > 0.05) (Figure 3D).

### Larval Survival, Growth, and Metamorphosis

During the larval planktonic period, the larvae underwent early-umbo, middle-umbo, and late-umbo and eye larval stages and then set the substrate-forming spat (Figures 4M–Q). Larval survival rate was 9.77% for the 2-month group, which was significant lower than those for the 3-month group (84.28%), 6-month group (82.15%), and 15-month groups (80.79%) (Figure 3E). The larval growth rate of the 3-month group (17.64 μm⋅d^–1^) was significantly higher than that of the 2-month (14.56 μm⋅d^–1^), 6-month (15.03 μm⋅d^–1^) and 15-month groups (14.00 μm⋅d^–1^) (*P* < 0.05) (Figure 3F). The metamorphic rate for the 2-month group was merely 3.45%, significantly less than that of the 3-month (72.91%), 6-month (70.63%), and 15-month groups (69.01%) (*P* < 0.05) (Figure 3G). The metamorphic time was 21.00 days for the 2-month group, 16.00 days for the 3-month group, 18.67 days for the 6-month group and 20.00 days for the 15-month group (Figure 3H). Metamorphic size ranged from 353 to 362 μm, with no significant differences between the experimental groups (*P* > 0.05) (Figure 3I).

### Spat Formation

Finally, 420, 55.36 × 10^4^, 52.75 × 10^4^, 50.46 × 10^4^ spat were obtained from about 90.00 × 10^4^ eggs from each of the 2-, 3-, 6-, and 15-month groups, respectively (Figure 4Q). Then, the spat underwent the youth and adult stages to complete their life history (Figures 4R–T).

## Discussion

### Gametogenesis During Early Life History

In this study, we only observed male sex differentiation in 1-month-old spat, because oysters are protandrous animals and exhibit rapid proliferation of spermatagonia (Hayes, 1998). Here, it should be pointed out that no female sex differentiation was observed due to the sample interval of 1 month. Since it is impossible to examine the gonad of individual at successive stages of its life, it becomes necessary to infer its history from an examination of other individuals at different ages (Coe, 1943; Yusa, 2007). At the physiologically mature stage, a large proportion of gametes are mature based on size and morphology, but are incapable of spontaneous spawning due to reproductive dysfunction until they enter the functionally mature stage.

The functionally mature stage of life history is defined as the age at which the female spontaneously produces the first batch of eggs (Collin, 1995; Llodra, 2002). Our results showed that the first functionally mature stage of the Kumamoto oyster started at 3 months old with a shell height of 9.0–12.0 mm. This opinion differs from the previous reports (Cáceres-Martínez et al., 2012), because they defined gamete maturity as the stage that is referred to as physiological maturity in this text. Age and size for functionally mature gametes could also be different among *Crassostrea* species. The shell height for first gamete maturity in *C. gigas* ranged from 16 to 39 mm (Buroker, 1983), while for *C. virginica* it is smaller than 35 mm, and as young as 42 days post-settlement (Buroker et al., 1979; Thompson et al., 1996). In summary, our re-examined results demonstrate that *C. sikamea* is the smallest species in the *Crassostrea* genus at functional maturity.

The sex ratio changed as the oysters grew, and there were significantly more males than females throughout the early gametogenesis process. Sex change is phylogenetically widespread but uncommon in both the plant and animal kingdoms (Policansky, 1982). Although it is rare among vertebrates, there is evidence of sex changes in most groups of invertebrates (Warner, 1975). Interestingly, we found the oocyte maturation of *C. sikamea* was delayed until 3 months old in this study. A small proportion of *C. sikamea* individuals in this study displayed hermaphroditism at 2 months old, but no hermaphrodites were observed at 3 months old, suggesting that this hermaphroditism may be occasional as intervening stage during early history stage (Guo et al., 1998; Hedrick and Hedgecock, 2010).

### Breeding Potential

To establish an effective oyster production management practices in a hatchery, it is very important to research gametogenesis in natural populations of bivalves in order to determine if the early development of gametes takes place in nature or if it was influenced by the hatchery operation (Cooper and Wintermyer, 2009). Our results indicate that no eggs can be obtained by dissection from spat with a shell height of 1.5–3.0 mm for hatchery experiment, despite expressed male sex differentiation. Moreover, presence of sperm in these oysters does not mean that they are physiologically active (Cáceres-Martínez et al., 2012). Once they reach a shell height of 3–5 mm, a small number of eggs (<1000/ind.) are partially functional; about 30% of eggs can be fertilized and 5% produce D larvae, with less than 4% forming spat. This indicated that, a large number of gametes from the physiological maturity stage are dysfunctional, leading to low fertilization, higher larval mortality and very low levels of metamorphosis.

When the shell height of *C. sikamea* reaches 9–12 mm at an age of 3 months, the oyster gonad becomes fully mature and contains eggs (>1 × 10^4^/ind.). We found that gametes from this stage were completely functional; almost 100% of eggs can be fertilized and produce viable progeny with high survival rate and grow fast. Therefore, this stage was defined as the functionally mature stage, which can spawn and produce viable progeny. Generally, the age of functional maturity plays a pivotal role in the life of an individual, dividing the lifespan of the organism into two phases, preparation and fulfillment (Stearns, 1992). This stage is extremely sensitive to age at maturity, because selection for age at first maturity results from the balance of benefits and costs of starting reproduction at that age (Cobo and Fransozo, 2005). This balance is affected by the interaction between age and size, which plays an important role in the determination of the onset of gametogenesis (Strathmann and Strathmann, 1982).

Our results clearly demonstrate that the early life history (fertilization, hatching, survival, growth, metamorphosis) of progeny from 3-month-old parents was similar to that of progeny from 6- and 15-months-old parents. However, the progeny from 3-month-old parents grew faster than those of the other three age parents. We speculated that the egg quality differed from other age parents, no spawning occurred in the 3-month-old oysters due to these functionally mature gamete comprising a large amount of endogenous nutrition (Loosanoff, 1962). In contrast, 6- and 15-month-old parents underwent spawning which may cause a lack of egg nutrition, leading to slow growth (Massapina et al., 1999). The Kumamoto oysters over 3 months old are possible to participate in reproductive activities in nature and help to restore the wild resource.

### Prospective Aquaculture Applications

Our results showed that first functional gametes from 3 months old parents can produce normal progeny, in other words, the 3 months old Kumamoto oyster is reproductively viable and produces healthy progeny, comparable to older broodstock tested in our study. Traditionally, 3–4 years old oyster were selected as parents in Taylor Shellfish Farm (United States), 2–3 years old oyster were selected as parents in Japan, 1–2 years old oyster were selected as parents in China (Zhang et al., 2017). Thus, our paper provides a new select standard of Kumamoto oyster parents, which is age 3 months old onward, where oyster size exceeds 9–12 mm shell height.

On the longer term, the successive selection of four generations for Kumamoto oyster by artificial select breeding currently requires 12–16 years in the United States (Personal communication), 8–12 years in Japan (Personal communication), and 4–8 years in China (Zhang et al., 2017). Based on our results, it is hypothetically to reduce the four generations breeding time for Kumamoto oyster to 12 months long, which is the great novelty in this study. One full generation of Kumamoto oysters only needs 3 months, which can shorten the breeding cycle and research biology traits as model organism in bivalves.

## Conclusion

Gametogenesis in *C. sikamea* began when individuals were very small and young, but most gametes were dysfunctional during the sex differentiation at 1 month old and physiological maturity life stages at 2 months old. Once the oysters reached 3 months of age, the gonad was full and contained a large number of eggs, which were completely mature and could spontaneously spawn. This was defined as the functionally mature phase. Successful artificial hatchery of *C. sikamea* was conducted using these viable gametes. No significant difference was observed among the different age groups on phenotypic traits of progeny from functionally mature gametes except growth ability. Results here provide the baseline data for the age selection of Kumamoto oyster parents during seedling production. Based on our results, we recommend to widen the empirical reproductive age of *C. sikamea* between 3 and 12 months old, with shell heights between 9–12 and 30 mm. This hatchery method can effectively shorten the breeding cycle, which also implies that these small oysters will participate in spawning and form a natural supplement for the oyster resource recovery.

## Author Contributions

ZY and YZ designed the experiments. ZY, LM, ZZ, and YP carried out all of the experiments with the help of YQ, SX, HM, and JL. JL analyzed the data and wrote the manuscript. ZY and YZ critically revised the manuscript and approved the final version to be published.

## Conflict of Interest Statement

The authors declare that the research was conducted in the absence of any commercial or financial relationships that could be construed as a potential conflict of interest.
